# Association of intestinal malrotation and Bochdalek hernia in an adult: a case report

**DOI:** 10.1186/1756-0500-7-296

**Published:** 2014-05-13

**Authors:** Raquel Salústio, Celso Nabais, Bárbara Paredes, Francisco V Sousa, Eusébio Porto, Caldeira Fradique

**Affiliations:** 1 Department of Surgery, Hospital de São José, Centro Hospitalar de Lisboa Central, Serviço de Cirurgia 1, Rua José António Serrano, 1150-199 Lisboa, Portugal

**Keywords:** Bochdalek hernia, Intestinal malrotation, Small bowel obstruction

## Abstract

**Background:**

Late presentations of congenital diaphragmatic hernia are rare and differ from the classic neonatal presentation. The association with other congenital malformations in children, mainly intestinal malrotation, is well documented. The diagnosis of this association in adults is very rare, and depends on a high degree of suspicion.

**Case presentation:**

We report a case of a 50-year-old female Caucasian patient with a previous history of intestinal malrotation diagnosed in adolescence and treated conservatively. She was referred to the hospital with signs and symptoms of intestinal obstruction. The patient undertook computed tomography that confirmed small bowel obstruction with no obvious cause, and a right subphrenic abscess with right empyema was also present. An exploratory laparotomy was performed that revealed an intestinal malrotation associated with a right gangrenous and perforated Bochdalek hernia. Resection of the affected small bowel, closure of the Bochdalek foramen and the Ladd procedure were carried out.

**Conclusion:**

This case shows a rare association of two rare conditions in adults, and highlights the challenge in reaching the diagnosis and management options.

## Background

Bochdalek hernia (BH) in adults is extremely rare, with less than 100 published cases in the literature (less than 50 were symptomatic) [1,2]. The clinical presentation with nonspecific gastrointestinal symptoms makes the diagnosis challenging, and so computed tomography (CT) is necessary for an accurate diagnosis.

Intestinal malrotation (IM) in adults is equally rare with an occurrence between 0.0001% and 0.19%. A low index of suspicion associated with the nonspecific clinical presentation commonly leads to delayed treatment [3].

Eighty percent of congenital diaphragmatic hernias (CDHs) in children are associated with other congenital malformations, mainly with IM (42% of cases), suggesting that these patients must be investigated [4,5].

## Case presentation

A 50-year-old Caucasian woman with previous history of IM diagnosed at 14 years of age, which had been followed-up with the general practitioner and had not been subjected to surgical intervention, was referred to the emergency department with an intestinal obstruction. She had a 3-day history of generalized abdominal pain, distension, vomiting and constipation. There was no previous history of trauma. Physical examination revealed a dehydrated patient with tachycardia, hypotension but no fever. Her abdomen was distended with mild hypogastric pain, but there was no guarding or rebound.

**Figure 1:**
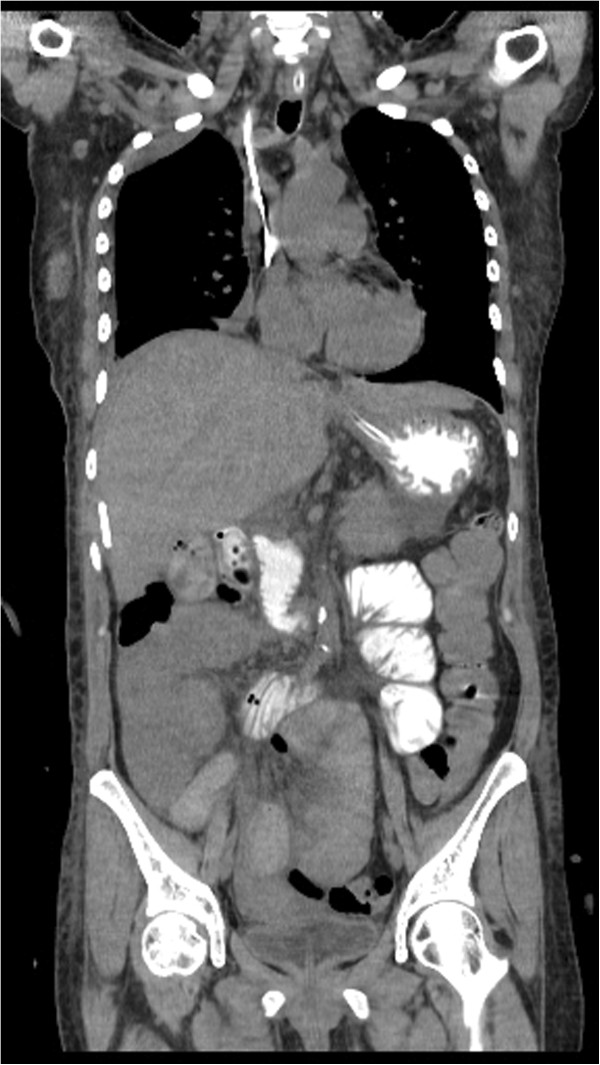
Computed tomography image (coronal view) showing small bowel obstruction associated with a subphrenic collection.

Laboratory testing showed leukocytosis with a white cell count of 15.8 × 10^9^/L (88% neutrophils), renal impairment with serum creatinine of 760.24 μmol/L (8.6 mg/dL), blood urea nitrogen of 84.61 mmol/L (237 mg/dL), and serum sodium was 130 mmol/L and potassium was 6.4 mmol/L. Chest X-ray showed an opacification in the right base. Abdominal CT with oral contrast revealed dilated and fluid-filled loops of the small bowel with an unidentified mechanical obstruction associated with a right subphrenic fluid collection. The exam showed also an atypical right pleural effusion compatible with an empyema (Figure 1).

**Figure 2:**
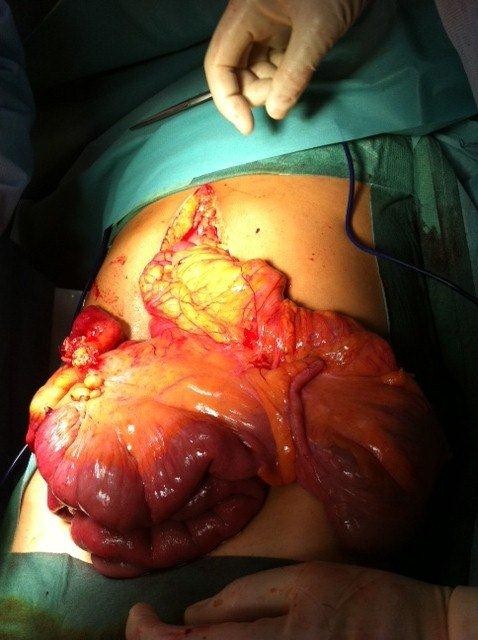
Intestinal malrotation.

**Figure 3:**
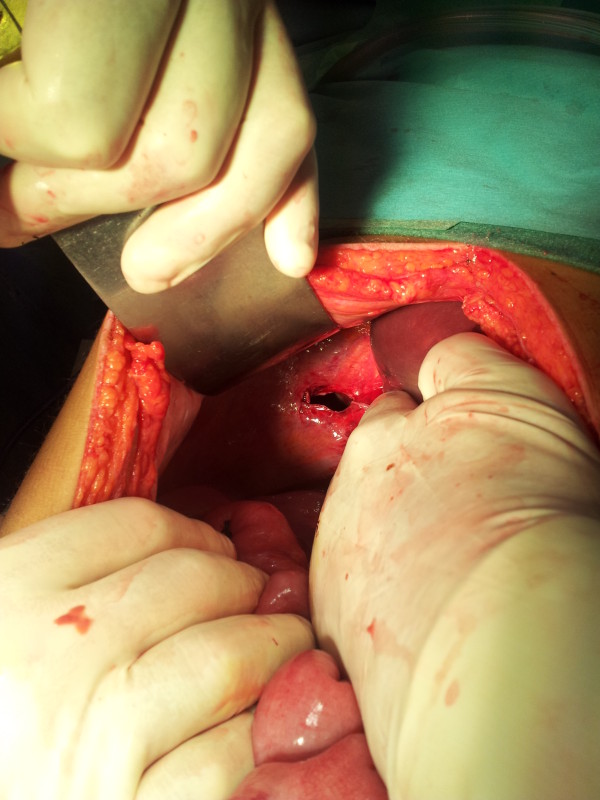
Right-sided foramen of Bochdalek after hernia reduction.

An emergency exploratory laparotomy was performed. There was fusion of the right and transverse mesocolon and the root was limited to the Treitz angle. The duodenum was in a right laterocolic location and the free cecum was located in the left hypochondrium, as well as the ascending and transverse colon (Figure 2). The right lobe of the liver was practically without its triangular ligament and was at a median location. We also found a right-sided BH with transdiaphragmatic, gangrenous and perforated ileum (Figure 3). The following procedures were carried out: reduction of the hernia content, resection of the ileal segment with entero-enteric anastomosis, closure of the right Bochdalek foramen, and the Ladd procedure.

During the first postoperative week, the patient stayed in the intensive care unit and made good clinical progress. She remained hemodynamically stable and with concomitant respiratory stability. A follow-up abdominal CT showed a right subphrenic air-fluid collection, which was percutaneously drained under ultrasound guidance. In the second postoperative week, there was an increase in the inflammatory parameters and the patient was diagnosed with a supradiaphragmatic collection that was drained under CT guidance.

The patient was discharged on the third postoperative week. No complications were identified in the subsequent follow-up checks.

## Discussion

Separation of thoracic and abdominal cavities occurs during the eighth week of gestation with the closure of the pleuroperitoneal canal. A failure in this process gives rise to CDH. The BH is a defect in the posterolateral closure of the pleuroperitoneal folds that gives rise to the diaphragm, and has an estimated incidence of 1 in 2000–5000 newborns [1,2]. The true prevalence is hard to estimate but the diagnosis of the condition has increased because of the availability of imaging in the investigation of non-specific symptoms [2]. The largest existing study estimated an incidence of 0.17% after a review of 13,138 CT scans; this incidence is notably similar to that found in newborns [6,7].

Described for the first time in 1848 by Bochdalek, it usually manifests in infancy with acute respiratory failure [1]. Larger defects are associated with pulmonary hypoplasia on the affected side and respiratory distress syndrome after birth. Minor defects are not associated with a deficit in lung development and may be asymptomatic until herniation of abdominal contents into the thoracic cavity with respiratory consequences. Several intra-abdominal organs can migrate through the diaphragmatic defect. Colon is the most common and results in large bowel obstruction [1,8-10]. Herniations of the stomach, small bowel, spleen, greater omentum and kidney have been described [11]. The risk of strangulation of herniated organs makes it a surgical emergency [1].

The signs and symptoms of this condition are non-specific and are more frequently related to the digestive tract (intermittent abdominal pain, vomiting and dysphagia) than the respiratory system (thoracalgia and dyspnea), thus differing from the classic neonatal presentation [1,2,4]. This late presentation is more frequent in men (3:1) and on the left side (70–90%), and is rarely bilateral or right-sided [1]. The diagnosis can be suggested by a chest X-ray with air-fluid levels or a supradiaphragmatic mass with costodiaphragmatic recess opacification and small pleural effusion [1]. CT is the main diagnostic tool. The differential diagnoses are hiatus hernia, pulmonary atelectasis, pericardial cyst and anterior mediastinal mass [1,2,12].

Definitive treatment is surgery with reduction of the herniated content and closure of the diaphragmatic defect.

IM is also a pediatric condition with 90% of cases diagnosed in the first year of life. The incidence is estimated to be 1 in 500 births, but the real incidence is difficult to determine because many are asymptomatic [3,13]. The incidence in adults seems to be increasing with the higher use of diagnostic imaging exams. The postmortem incidence of IM is estimated to be up to 1 in 6000 adults [3].

Physiologic herniation of the midgut through the umbilical cord occurs during the fourth to fifth week of gestation, and through to the ninth to tenth week when it returns to the abdominal cavity attached to the retroperitoneum. During this process, a 270° anticlockwise rotation around the upper mesenteric artery occurs, resulting in duodenal arch formation, later emerging as the Treitz ligament (TL). The duodenojejunal loop shifts to the left, and the distal small bowel shifts progressively to the right; the cecum descent completes the rotational process. Finally the mesentery adheres to the retroperitoneum diagonally from the TL to the cecum [13].

IM is a result of anomalies in its location and fixation, which varies between normal, incomplete, reverse and nonrotation.

Incomplete rotation is the most frequent and occurs when there is a partial rotation of the duodenum and the right colon. The proximal portion of the midgut rotates 90° and the distal portion rotates 180°, placing the duodenum on the right side of abdominal cavity and the cecum under the stomach. It results in a shorter distance between the TL and the cecum, and consequent narrowing of the mesentery root [13]. A thickened band of mesentery (Ladd’s bands) can join the cecum to the duodenum and tighten the latter. If a large portion of intestine is left suspended from the posterior abdominal cavity with only one point of fixation, it is prone to torsion and formation of a volvulus.

Nonrotation or atypical rotation is the most frequent in adults, and results from abnormal intestinal positioning and inappropriate fixation of its root, which becomes short [7]. The midgut fails to complete its final rotation of 180° even though the first 90° rotation occurs normally. This results in the proximal portion (mainly the colon) remaining on the left side of the abdominal cavity and the distal portion (mainly small bowel) being located on the right. The TL is in the middle or on the left, below the pylorus, with or without a mobile cecum [13].

Presentation with chronic symptoms is the most common form and 70% of patients have symptoms for six months or more before the diagnosis is made, with vague or intermittent abdominal pain, nausea, vomiting, diarrhea, premature satiety and abdominal distension [5]. The acute presentation with intestinal obstruction due to a volvulus is less frequent, which is different from that seen in childhood [3]. It is important to note that a low index of suspicion in adults leads to an incorrect diagnosis even in their acute forms, in contrast to children where the diagnosis is correct in over half of cases preoperatively (57%) [5].

Imaging contrast studies can reveal a vertical duodenum, lack of duodenojejunal flexure (present in 80% of cases), abnormal location of cecum or colon. These exams have been replaced by CT scan [3,13,14]. CT scan allows the evaluation of superior mesenteric vessels and the position of the duodenum, atrophy or absence of the uncinate process of pancreas, and the “whirlpool appearance” of the small bowel. This was first described by Fisher, and represents the volvulus of the midgut around the vascular pedicle caused by its narrowed root [3].

The surgical treatment for typical IM is the universally accepted Ladd procedure—sectioning of the Ladd bands, widening of the mesentery, appendectomy and intestinal repositioning [13]. The treatment of atypical IM is less defined in both children and adults because of the predominance of chronic symptoms and the significant percentage of asymptomatic individuals [13]. It is known that these have a low risk of intestinal volvulus and regular follow-up is recommended. This expectant management is supported by the risk of postoperative complications (60%) and need for re-intervention [5,13]. On the other hand, it is impossible to predict when acute complications will arise.

Evidence suggests that the association between IM and CDH is related to an abnormal positioning and intestinal fixation in an abdomen that has a large communication with the thorax, and the right and left lobes of the liver can be absent [4,7,15]. Evidence also suggests that the existence of CDH permits displacement of the abdominal viscera into the thoracic cavity, thus distorting the intestinal anatomy [15].

## Conclusion

This pathologic entity is rarely reported in the literature and the diagnosis is usually delayed with consequences. Other congenital malformations need to be excluded and close follow-up of cases diagnosed in the first years of life is important.

Management of asymptomatic cases of IM is not linear.

Clinical history focusing on dyspeptic symptoms (frequent in the general population) and imaging with oral contrast are fundamental for establishing the diagnosis.

Surgeons must be alert to the possibility of emergency surgery in these rare cases.

## Consent

Written informed consent was obtained from the patient for publication of this Case Report and any accompanying images. A copy of the written consent is available for review by the Editor-in-Chief of this journal.

## Abbreviations

BH: Bochdalek hernia; CT: Computed tomography; IM: Intestinal malrotation; TL: Treitz ligament.

## Competing interests

The authors declare that they have no competing interests.

## Authors’ contributions

CN, BP, FS, EP and CF analyzed and interpreted the patient data, and contributed to the design and revisions of the manuscript. RS analyzed and interpreted the patient data, wrote the manuscript, and obtained informed consent. All authors read and approved the final manuscript.
